# Traumatic Globe Luxation and Optic Nerve Avulsion: A Case Report and Literature Review

**DOI:** 10.7759/cureus.53150

**Published:** 2024-01-29

**Authors:** Kevin Kwan Joo Ern, Julieana Muhammed, Nurul Huda Amin, Safiyah Jameelah

**Affiliations:** 1 Ophthalmology, Universiti Sains Malaysia, Kota Bharu, MYS; 2 Ophthalmology, Melaka Hospital, Malacca City, MYS

**Keywords:** enucleation, motor vehicle accident, le fort fracture, optic nerve avulsion, globe luxation

## Abstract

Traumatic globe luxation associated with optic nerve avulsion is rare. We describe a case of a 42-year-old Indian gentleman who was involved in a motor vehicle accident (MVA). He sustained a deep laceration wound from the right side of the nose extending to the left medial canthal region and left eyelid. The left globe was not visualized, and only the left optic nerve stump was seen. A computerized tomography (CT) scan showed a left globe dislocated inferotemporal with discontinuity of the left optic nerve, inferior rectus, and lateral rectus muscle. There were also comminuted fractures at the floor and lateral wall of the left orbit with significant lateral displacement of the bone fragments. He underwent left eye enucleation, toilet, and suturing of the laceration wounds and open reduction internal fixation (ORIF) of the fractures. We report this case as it is not commonly seen in Malaysia.

## Introduction

Traumatic luxation of the globe with optic nerve transection is rare [1]. It is more commonly seen in males with a mean age of 29.5 years [1]. It is defined as the complete or partial displacement of the globe from the orbit [2]. Clinicians should suspect associated optic nerve avulsion if a patient presents with globe subluxation or dislocation with vision loss in blunt or penetrating trauma to the globe [3]. It is commonly due to high-energy trauma such as motor vehicle accident (MVA) [4]. Here, we describe an individual who presented with globe luxation associated with optic nerve avulsion following an MVA.

## Case presentation

This is a case of a 42-year-old Indian gentleman with underlying diabetes mellitus and hypertension. He presented to the Emergency Department (ED) after being involved in an MVA that involved two cars. He was one of the car drivers, and the exact mechanism of the accident was not known.

**Figure 1:**
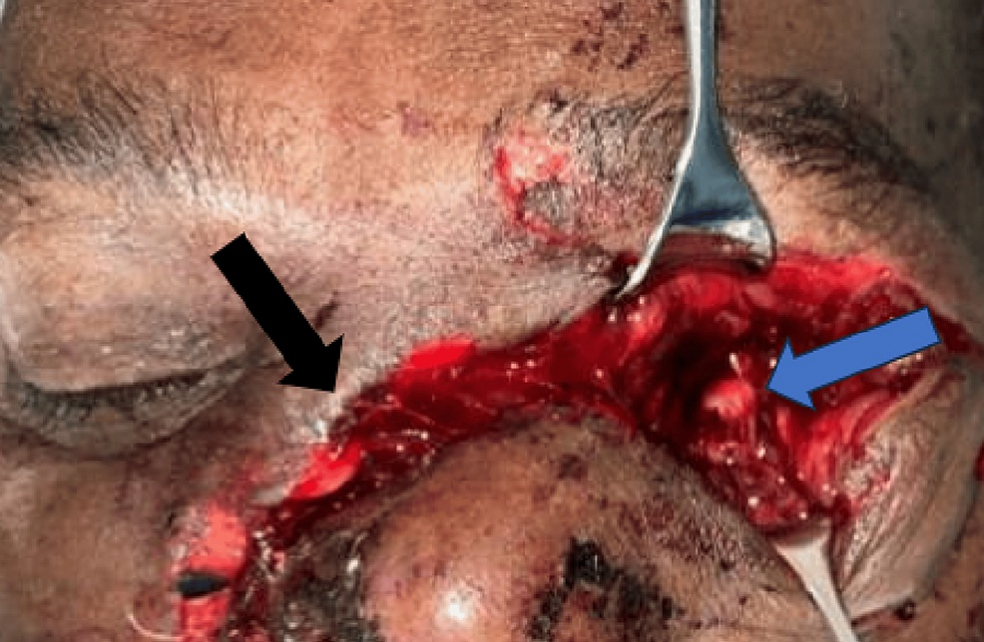
Intraoperative view showing left globe missing with only optic nerve stump seen (blue arrow). There was a deep laceration wound from the right side of the nose extending to the left medial canthus and the upper and lower left eyelid (black arrow)

His visual acuity was no light perception, and 6/12 for the left and right eye, respectively. The left globe was not visualized; only the optic nerve stump was seen within the orbital cavity (Figure 1). There was also a deep laceration wound from the right side of the nose extending to the left medial canthus and the upper and lower left eyelid (Figure 1).

**Figure 2:**
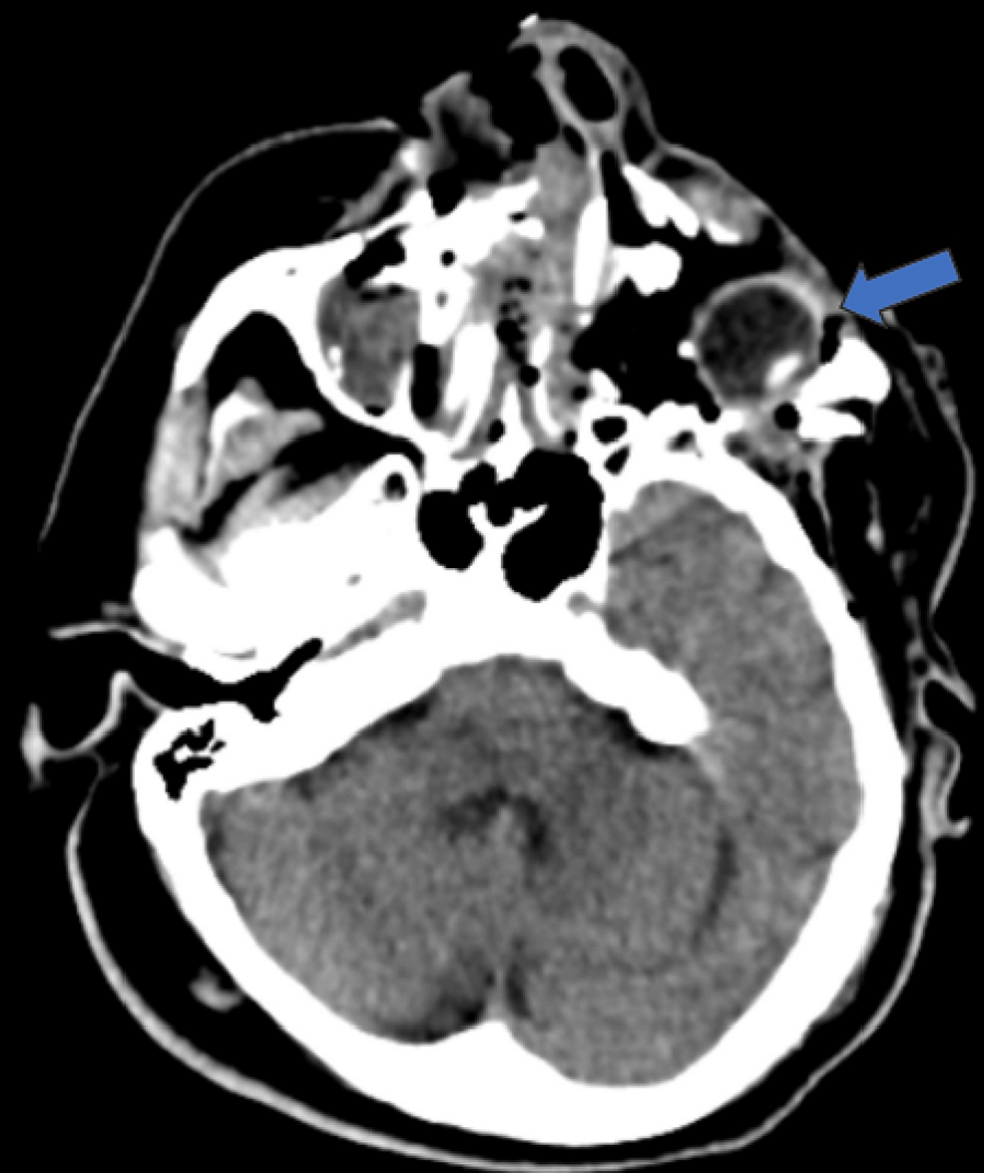
CT scan showing left globe luxation inferotemporally (blue arrow). The globe was intact.

**Figure 3:**
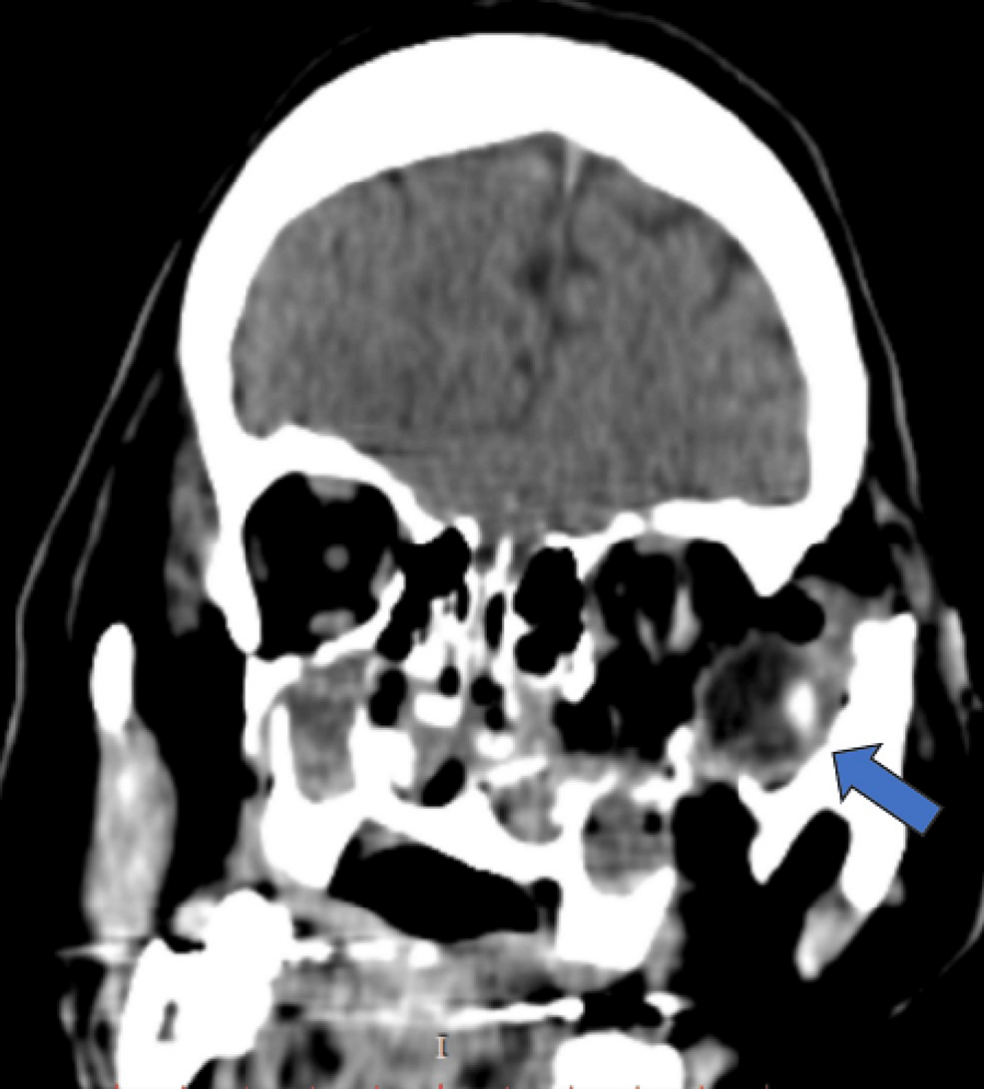
CT scan (coronal view) showing left globe luxation inferotemporally (blue arrow) with fracture over the floor and the lateral wall of the left orbit.

CT scan revealed a comminuted fracture of the floor and lateral wall of the orbit with significant lateral displacement of the bone fragments. Depressed bone fragments from the fractured inferior orbital rim into the orbit were also seen. There was complete luxation of the globe inferotemporally (Figures 2, 3) with discontinuity of the left optic nerve, inferior rectus, and lateral rectus muscle. He also sustained a Le Fort Ⅲ fracture, left zygomatic fracture, naso-orbitoethmoid complex fracture, nasal bone, and septum fracture, and closed fracture of the right radius and ulna.

**Figure 4:**
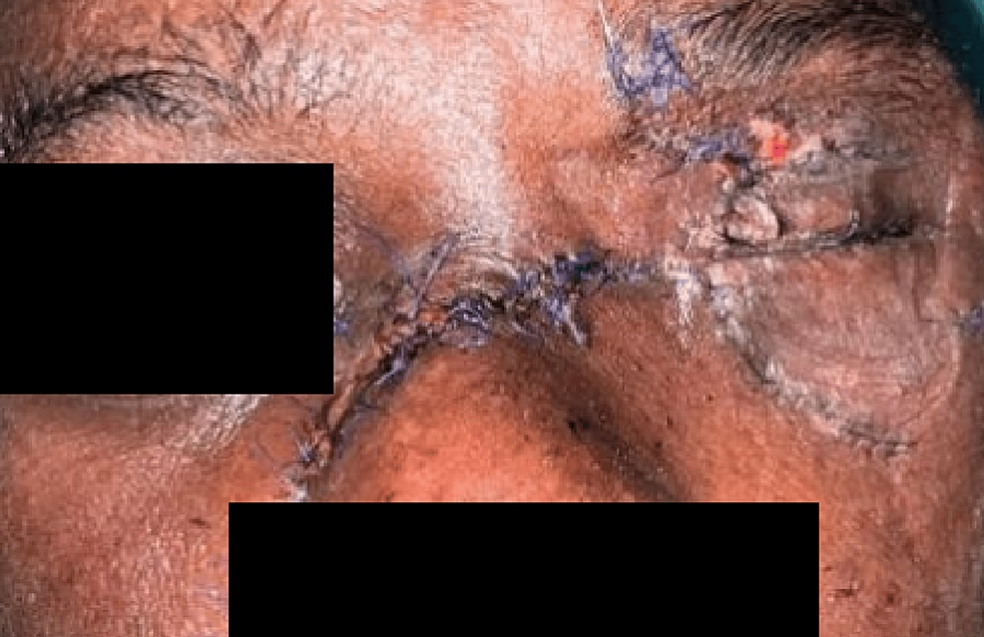
Post left eye enucleation, toilet, and suturing of laceration wound and left temporary tarsorrhaphy.

He was co-managed by OMFS (Oral and Maxillofacial Surgery), PRS (Plastic and Reconstructive Surgery), ORL (Otorhinolaryngology), and the Orthopaedic team. He underwent left eye enucleation, open reduction, and internal fixation (ORIF) of the left zygomatic complex, bilateral maxilla and naso-orbitoethmoid complex fractures, toilet, and suturing for the laceration wounds with left eye temporary tarsorrhaphy the next day, where the procedure took 6 hours. Postoperatively, he recovered well, and no wound gapping was seen (Figure 4). Unfortunately, the patient passed away on post-trauma day three due to an extensive pulmonary embolism.

## Discussion

There are two types of globe luxation. The first type is when the globe is expelled from the orbit. The second is when the globe dislodged into the paranasal sinuses [1]. Morris et al describe three theoretical theories behind globe luxation. The first theory is when a long object enters the medial orbit and uses it as a pivot to propel the globe forward. In the second theory, he describes a wedge-shaped object that enters the orbit medially and pushes the globe anteriorly by pressuring the eye against the lateral wall of the orbit until it exceeds the intraorbital pressure. The third theory is when there is a direct laceration of the optic nerve by a sharp object [5]. Kreiner et al. theorized that globe luxation into the paranasal sinuses is due to the mechanism of blowout fracture [6], where the object in question is larger than the orbital rim- for example, such as fists and dashboards [7]. The strong, blunt force fails to damage the globe as it has numerous protective structures, such as a tough orbital rim, facial tissue, extraocular muscles, ligamentous tissue, and orbital fat [7]. However, although the globe is not damaged, the force is strong enough to dislodge the globe through the thin orbital wall [6]. Among the paranasal sinuses, the maxillary sinus is most commonly affected [1]. This is due to its large size and location close to the orbital cavity [8]. Statistical data from Kreiner et al. shows that direct orbital trauma is the most common cause of traumatic globe luxation with associated fractures of the medial and floor of the orbital wall [6].

Globe luxation with associated optic nerve avulsion is also not commonly seen due to the tortuous intraorbital portion of the optic nerve, which allows room for maneuvering. When it happens, it is likely due to a few reasons. The first is the forward thrust of the globe, causing associated optic nerve avulsion. The second is due to the sudden rise in intraorbital pressure, and the third is due to direct trauma to the optic nerve [8].

In this case, the patient had an inferotemporal globe luxation with an associated optic nerve avulsion. Even though there was an associated maxillary wall fracture, the globe was displaced inferotemporally through the lateral orbital wall. Further history revealed that the patient had many items on the car dashboard, such as cigarettes and lighters, which could contribute to his injuries. We postulated it could be a combination of mechanisms described by Kreiner et al. where the object is larger than the orbital rim, causing globe luxation [6]. This, along with Morris et al.’s and Burns et al.’s theory, where there is a direct optic nerve transection due to penetrating injury [5,8]. It is supported by his clinical presentation of a deep laceration wound from the right side of the nose extending to the left eyelid.

In an ideal situation, if the globe is intact, preservation and repositioning are the first treatments of choice [1]. This provided the patient with better functional, aesthetic, and psychological outcomes, especially when facing this traumatic event [4]. However, the management is controversial if there is associated optic nerve avulsion. Fortunately, there was no reported sympathetic ophthalmia related to these injuries [9]. However, given the nature of such injuries, we must continue to observe them in the fellow eye [10]. Lelli et al. felt globe repositioning should be offered before considering other options like enucleation [9]. Despite that, the patient still had to undergo enucleation two months later [9]. This is similar to a case reported by Amaral et al., where there was pain and unsatisfactory cosmetic results [1]. A paper by Chin et al. showed that early placement of ocular prostheses provides satisfaction to patients [11]. In this case, we proceeded with enucleation with plans for an orbital prosthesis early in the future after discussions with the patient and his family members. There are a few cases of traumatic globe luxation which has been reported for the past five years, as listed (Table 1) [2,3,10,12-16]. In certain situations, enucleation and prosthesis implantation were the treatments of choice, while in others, globe repositioning was the preferred treatment.

**Table 1:** Summary of reported cases of traumatic globe luxation for the past five years

Author (year)	Methodology (case report)	Mechanism of injury	Findings and associated injury	Optic nerve avulsion	Management	Outcome
Savur et al. (2022) [12]	Case report	Motor vehicle accident	Full thickness skin defect from left upper lid and extending to the scalp in the frontotemporal region. Left globe luxation. CT imaging shows fracture over bilateral frontal bone and medial and lateral orbital wall.	Not available (NA)	Globe repositioning	Phthisis bulbi.
Omari et al. (2022) [3]	Case report of two cases	First case: Motor vehicle accident	Sustained periorbital hematoma and superficial eyelid lacerations. Left globe luxation. CT imaging results was not available.	Yes	Enucleation	Prosthesis implanted
		Second case: Motor vehicle accident	Left eyelid laceration wound. Left globe luxation. CT imaging shows fracture of the orbital floor	Yes	Enucleation	Prosthesis implanted
Shafa et al. (2022) [10]	Case report	Head trauma	Left facial soft tissue injury with hematoma at the left temporal side of her head. Left globe luxation. CT imaging shows left orbital wall dislocated to the posterior-lateral side	Yes	Enucleation	Not available
Meena et al. (2020) [2]	Case report	Motor vehicle accident	Full thickness laceration wound over the left upper eyelid. Left globe luxation. CT imaging shows fracture of the floor, medial and lateral wall of the left orbit	Yes	Enucleation	Not available
Das A et al. (2020) [13]	Case report	Motor vehicle accident	Edema and ecchymoses over right side of the face. Right globe luxation. CT imaging shows communited fracture of the frontal bone extending into the roof and lateral wall of the right orbit.	Yes	Enucleation	Not available
Elkbuli et al. (2020) [14]	Case report	Assault	Right lower lid laceration. Right globe luxation. CT imaging shows diffuse gas within the right orbit and disruption of the intra-orbital structures	Yes	Enucleation	Prosthesis implanted
Das D et al. (2019) [15]	Case report	Cow horn injury	Extensive laceration wound of the left eyelid. Left globe luxation. CT imaging shows globe luxation with optic nerve avulsion	Yes	Globe repositioning	Left eye exotropia with hypertropia
Gaur N et al. (2019) [16]	Case report	Assault with iron rod	Full thickness lid laceration involving lid margin of left upper lid with globe luxation. CT findings shows complete transection of optic nerve along with disinsertion of left medial rectus from insertion. No orbital wall fractures	Yes	Globe repositioning	Left eye exotropia.
Our case	Case report	Motor vehicle accident	Deep laceration wound from the right side of the nose extending to the left medial canthus and the upper and lower left eyelid. CT findings show complete luxation of the globe inferotemporally with discontinuity of the left optic nerve, inferior rectus, and lateral rectus muscle. There was communited fracture of the floor and lateral wall of orbit	Yes	Enucleation	Not available

## Conclusions

Traumatic globe luxation with optic nerve avulsion is not commonly encountered. Early surgical attempts to reposition the globe would provide better psychological and cosmetic benefits. However, it is essential to discuss the options available with the patient and family members, such as globe repositioning or early enucleation with an ocular prosthesis.
